# Recovery of Valuable Materials and Methods for Their Management When Recycling Thin-Film CdTe Photovoltaic Modules

**DOI:** 10.3390/ma14247836

**Published:** 2021-12-17

**Authors:** Anna Kuczyńska-Łażewska, Ewa Klugmann-Radziemska, Agnieszka Witkowska

**Affiliations:** 1 Department of Energy Conversion and Storage, Faculty of Chemistry, Gdansk University of Technology, G. Narutowicza Str. 11/12, PL-80-233 Gdańsk, Poland; ewa.klugmann-radziemska@pg.edu.pl; 2 Institute of Nanotechnology and Materials Engineering, Faculty of Applied Physics and Mathematics, Gdansk University of Technology, G. Narutowicza Str. 11/12, PL-80-233 Gdańsk, Poland; agnieszka.witkowska@pg.edu.pl

**Keywords:** photovoltaic CdTe modules, recycling, metals recovery, renewable energy sources

## Abstract

Due to the development of new photovoltaic technologies, there is a need to research new recycling methods for these new materials. The recovery of metals from photovoltaic (PV) modules would reduce the consumption of raw materials. Therefore, the development of recycling technologies for used and damaged modules of newer generations is important for environmental reasons. The aim of the research is to shed light on the nature of the chemical reactions that occur in recycling technology for second-generation photovoltaic modules. This work is focused mainly on cells made of Cadmium Telluride (CdTe). It was proven that prior thermal delamination was necessary. Moreover, an improvement in understanding of the recovery process depending on used leaching solution was achieved.

## 1. Introduction

Modules based on crystalline silicon and modules made with thin-film technologies must be recycled in three stages: delamination, material removal, and material recovery. In both cases, delamination plays a key role in the recycling process. However, for the thin-film modules, material removal and recovery are harder because it is not possible to separate the cell plates and reuse them [1]. It is a moot point whether recycling of the thin-film modules is profitable because the semiconductor material constitutes less than 1% of the cell mass [2].

The yields for glass and semiconductor recovery during CdTe recycling processes exceed 90%, and literature studies mention that this yield could possibly reach 99.99% for Cd by using ion-exchange resins. [3]. Instead of disposal, recycling is now the favoured option for the management of end-of-life CdTe photovoltaic modules. The unintentional emission of Cd into the environment can therefore be avoided. The amount of Cd in one module is not significant, but collectively in the photovoltaic waste it is an environmental problem. 

The variety of technologies used in the production of thin-film cells require different recycling approaches; therefore, for indium copper gallium diselenide (CIGS) and cadmium telluride (CdTe) modules, pyrolysis followed by chemical treatment is appropriate, whereas for modules based on amorphous silicon (a-Si), the first stage is grinding, followed by the pneumatic separation of the polymer base [4].

### 1.1. Thin-Film Cell Production

Amorphous silicon is a non-crystalline allotrope obtained from silicon and is used not only for the production of photovoltaic cells, but also for LCD and OLED screens. This material is produced from a mixture of gases (SiH_4_ and PH_3_/B_2_H_6_) in a plasma furnace under subpressure and is deposited on the base. This technology enables a decrease in the amount of used material and the production of a large cell surface simultaneously. The structure of a-Si cells differs from the structure of crystalline silicon cells because there is a p-i-n and n-i-p joint. In this junction, there is a layer of undoped silicon. The feature that significantly distinguishes thin-film cells from crystalline silicon cells is the operating temperature being inversely related to the efficiency. Although the efficiency initially increases as the temperature increases, after irradiation, it decreases and stabilizes at a lower level.

In the case of CdTe cells, the front contact comprises indium tin oxide (ITO), while the next layer is n-type cadmium sulfide and p-type CdTe, acting as an absorber. The rear metallic contact is created by spraying. CdTe seems to be a promising material for the production of solar cells due to its high solar absorption coefficient.

Copper indium diselenide (CIS) is also increasingly used because it also has a high optical absorption coefficient and low resistivity. Thin CIS layers are obtained by evaporation at a high temperature (1300 °C) or by cathodic sputtering [5]. It is also possible to deposit two-component compounds or individual elements from various sources by vacuum evaporation.

### 1.2. Recycling Process

To prevent the leaching of harmful substances from the panels, insulation technologies are used, but these impede the recycling process. Delamination is the biggest issue when recycling PV modules. The simplest solution is to grind entire modules to obtain small fragments that are suitable for further processing [2]. There are also innovative solutions, such as the microemulsion method, that have been implemented in Germany. However, the most common method is pyrolysis at temperatures in the range of 300–600 °C, as this approach allows the complete removal of organic material. This subject is discussed in more detail in a previous work [6].

An overview of the recycling methods for solar modules is presented in Table 1. For the description of the recycling technology scale, three terms were used. “General” was used for methods generally used in the industry on a large scale by companies. “Academic research” was used for methods in the early stages of development, made in the laboratory conditions. “Pilot studies” was used for patented solutions and big scientific projects in cooperation with the industry

The initial methods were focused on acidic leaching with acids such as nitric acid [7] or sulfuric acid mixed with hydrogen peroxide [4]. Currently, the limitation of the consumption of harmful chemicals plays a key role. One concept is the direct removal of the semiconductor layer by tracing, after initial thermal delamination [8]. However, the most promising method from an environmental point of view is wet separation with water; a recovery of CdTe of 94% can be obtained by screening [9].

The next step in the recycling process is the recovery of the washed metals. This can be achieved by the electrolytic separation of the metals into individual fractions [7]. However, research has begun on less demanding methods such as precipitation with calcium oxide or carbonate, and calcination of the resulting sludge [10] or precipitation with sodium hydroxide and thickening of the resulting sludge [4]. However, these methods generated a significant amount of waste and demand for electricity.

An example of a recycling technology for thin-film cells based on CdTe [13] is mentioned in the Table 1. In the first stage, the modules are crushed, then washed with a solution of sulfuric acid (VI) and hydrogen peroxide. A solution rich in cadmium, tellurium, copper, and iron ions is obtained. To separate the copper, columns with a chelating agent are used, and cadmium and iron are separated in a cation-exchange column. The final release of cadmium occurs during an electrochemical process. The tellurium is precipitated from the solution as an oxide with sodium carbonate.

The technology of recycling photovoltaic cells based on CIGS [7] is presented in Table 1. After crushing, modules are subjected to a washing process with nitric acid (V). Metals, such as indium, selenium, copper, and zinc, pass into the solution in oxidized form, while tin oxide (SnO_2_) remains on the glass layer. The hydrolyzed EVA begins to float on the surface of the washing solution, from where it will be easily removed.

The metals are recovered from the solution during the DC electrolysis process. An appropriate selection of process parameters and electrodes allows for the two-stage separation of the Cu/Se and Cd mixtures. The oxidation and distillation processes separate copper and selenium in the form of oxides.

The recycling process used by First Solar consists of grinding modules and then removing the laminates by bathing them in 30% hydrogen peroxide. Liquid–solid separation occurs using centrifuges and vibrating screens, and metal recovery is accomplished by precipitation and filtration. This process significantly reduces the negative impact of used thin-film modules with CdTe, for example, by reducing the total energy demand from 81 MJ/m^2^ to 12 MJ/m^2^ [14]. It is also possible to reduce the harmful impact of this type of module on the environment by about 10% in categories such as: general energy demand, acidification, eutrophication, global warming, and photochemical ozone hole creation [14].

A novel approach, such as the use of energy-efficient smart controls during thin-film CdTe thermal treatment processes [15], also helps to reduce the harmful impact of the new technologies. Photovoltaic modules based on CdTe are treated in dedicated recycling plants integrated with module production plants, where the semiconductor materials are recovered in addition to glass and copper [3]. The recovered materials are directly used in manufacturing processes.

## 2. Materials and Methods

Samples of CdTe thin-film modules made by Advanced Solar Power Hangzhou INC (Hangzhou, China) were milled in a Pulverisette 9 vibratory disc mill from Fritsch GmbH (Idar-Oberstein, Germany). The samples were milled for 10 min at 1500 rpm. After milling, samples were sifted and no grains with a size above 1 mm were present. Most of the sample grains were less than 100 μm. Thermal decomposition was performed in a horizontal tube furnace PTF 12/105/500 (Protherm Furnances, Ankara, Turkey) equipped with a PC 442/MP20 controller. The samples were placed in a crucible and treated at 600 °C for 3 h according to previous research [6]. The rate of the temperature increase in the furnace was 10 °C/min.

Then, samples (1 g) were placed in 30 mL of the following three types of the most commonly used etching solutions: 1 M H_2_SO_4_, 3 M HNO_3_, and 30% H_2_O_2_. The etching process was conducted at a constant temperature in a water or oil bath, at 30 °C and 50 °C, respectively. After the leaching process, the sample was filtered under vacuum. After drying and weighing the precipitate, the absolute weight loss was calculated.

The surface composition of the powders after the leaching process was determined by X-ray photoelectron spectroscopy (XPS) using an Omicron XPS spectrometer system (ScientaOmicron, Uppsala, Sweden) operating at a base pressure of 2 × 10^−9^ mbar, and with achromatic radiation from a Mg anode. A scan step and pass for high-resolution scans with energy equal to 0.02–0.05 eV and 50 eV, respectively, were used.

The effects associated with the charge loading of samples were removed by calibrating to the carbon C1s band (284.5 eV). Elemental composition analysis and decomposition of the obtained bands were conducted using the NIST XPS Database [16] and the XPSpeak software [17]. The Shirley background and Gaussian–Lorentz product function were used to fit the C1s, O1s, Si2p, Te3d, and Cd3d bands. For quantitative analysis, the following atomic sensitivity factors were applied: ASF(O1s) = 0.63, ASF(Si2p) = 0.17, ASF(Cd3d5/2) = 2.55, and ASF(Te3d5/2) = 4.0 [18].

All analyses were performed in order to find out the chemical composition on the surface of the solid samples before and after the etching process.

## 3. Results and Discussion

### 3.1. Leaching Process Results

**Figure 1:**
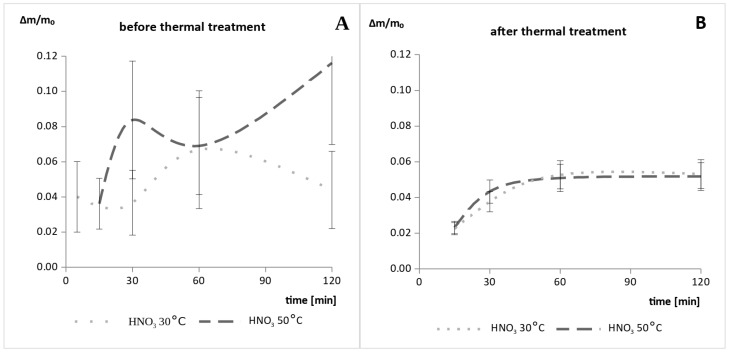
Etching results for samples of the milled thin-film module made of CdTe leached in 3 M HNO_3_ at 30 °C and 50 °C (**A**) before and (**B**) after the thermal delamination process.

Attempts to etch the milled fragments of the thin-film module made of CdTe confirmed the need for additional thermal delamination after the mechanical delamination process. Samples that were not baked after grinding contained residuals of the laminating material despite screening, which caused the sticking of the mixing system components that adversely impacted the measurements of the weight loss (Figure 1).

**Figure 2:**
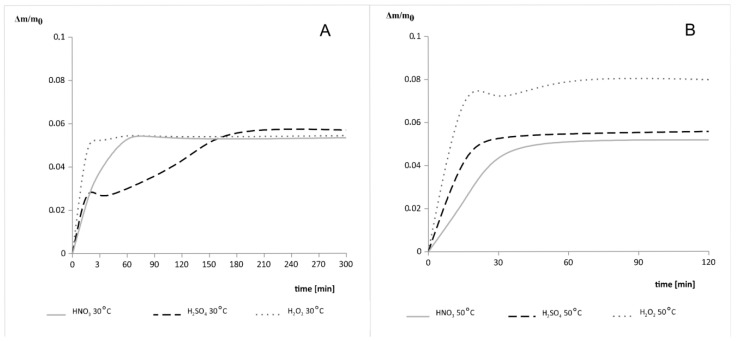
Etching results for samples of the milled thin-film module made of CdTe in 1 M H_2_SO_4_, 3 M HNO_3_, and 30% H_2_O_2_ at (**A**) 30 °C and (**B**) 50 °C.

After the samples were decomposed at 600 °C for 3 h, this problem was eliminated and no sticking of the system elements was observed (Figure 1B). Figure 2 shows the etching results for the samples of the milled thin-film module made of CdTe. The results obtained after the leaching in the 3 M HNO_3_ are similar regardless of the process temperature used. However, after etching in 1 M H_2_SO_4_, the weight loss during the process carried out at 50 °C was five times faster than the one carried out at a lower temperature. On the other hand, for the 30% H_2_O_2_ solution, an increase in total weight loss by one third was observed after increasing the process temperature.

### 3.2. Surface Composition

To confirm that the weight loss was related to the removal of cadmium and tellurium from the powder, XPS measurements and analysis were performed. The energy-dispersive X-ray (EDX) spectroscopy analysis did not allow the identification of the semiconductor material, because cadmium and tellurium were present in the sample in amounts within the error limit. This is consistent with the literature because a significant part of the sample was a matrix composed mainly of glass, which constituted over 95% of the mass composition of the module, whereas the semiconductor material was comprised from 0.16–0.18% [2].

**Figure 3:**
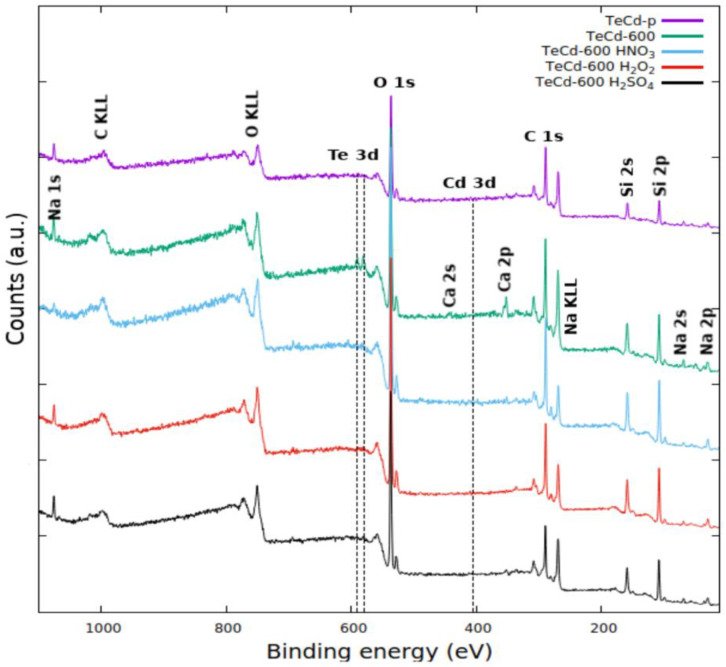
XPS survey-scan spectrum of samples before (TeCd-p) and after thermal treatment (TeCd-600) and after leaching for 2 h at 50 °C in 1 M H_2_SO_4_ (TeCd-600 H_2_SO_4_), 3 M HNO_3_ (TeCd-600 HNO_3_), and 30% H_2_O_2_ (TeCd-600 H_2_O_2_).

**Figure 4:**
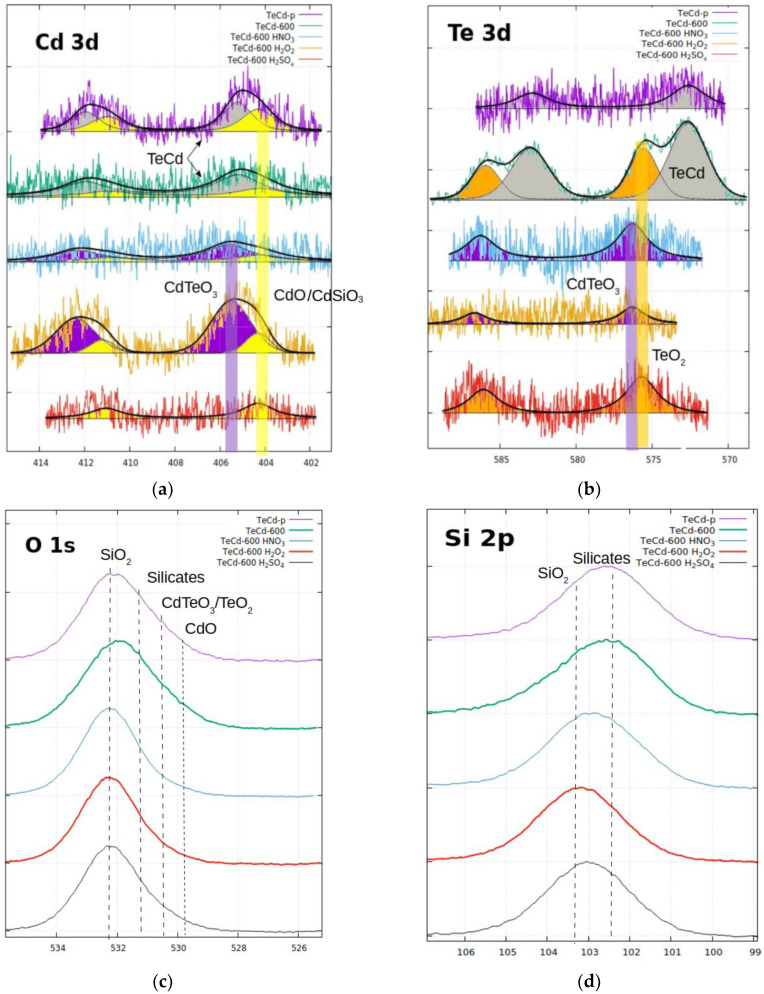
High-resolution XPS spectra measured at (**a**) Cd 3d, (**b**) Te 3d, (**c**) O 1s, and (**d**) Si 2p bands of all samples presented in Figure 3.

Only the XPS surface analysis allowed the observation of the traces of cadmium and tellurium in the samples (see Figure 3) and the study of their chemical environment by the collection and analysis of the high-resolution Cd 3d and Te 3d spectra (Figure 4).

The research conducted at the atomic-structure level regarding the recycling of thin-film modules allowed for the analysis of the phenomena that occurred during the recycling process.

In Figure 4, it can be seen that, before the thermal delamination process, the cadmium 3d lines are clearly visible, whereas the tellurium spectrum is hardly detectable. Cadmium was present in the form of cadmium oxide and CdTe, and tellurium was only present as CdTe, as suspected. After the thermal decomposition at 600 °C, the tellurium 3d lines become more intense and show the presence of CdTe and tellurium oxide, whereas the cadmium band stays without a change. Thus, the thermal decomposition removed the remains of the organic foil from the surface of the material, and tellurium oxide appears on the surface of the sample grains, as can be noted on the Te 3d spectra. Moreover, another important effect can take place at elevated temperature (above 500 °C)—the acceleration of the cadmium oxidation in air (k = 1.0·10^−9^ [g^2^/(cm^4^s)]) [19], resulting in the formation of CdO, which, above 600 °C, can react with silicon oxide and form silicates (shift of the O1s band maximum in Figure 4).

The XPS results for the Cd 3d band in Figure 4 confirmed the presence of cadmium in the form of oxide and/or silicates (in this band, it was difficult to separate both contributions). However, the presence of silicates before the leaching process was clearly confirmed with the XPS results collected around the energy of the Si 2p band and is shown in Figure 4.

The presence of silicates may explain the faster increase in weight loss after increasing the etching temperature with sulfuric (VI) acid (Figure 2).

Additionally, the silicates dissolve much more easily in the solution of sulfuric acid (VI) than pure cadmium (after leaching in 1 M H_2_SO_4_, the Cd 3d band practically disappeared). The increased total weight loss during leaching with the 30% hydrogen peroxide can be explained by the formation of telluranes in a highly oxidizing environment due to their reducing nature. Increasing the temperature of the process may stimulate the reaction. According to the Si 2p, Cd 3d and Te 3d bands shown in Figure 4, it can be assumed that, after leaching in the 30% H_2_O_2_ solution, silicates present in the sample transform into SiO_2_ and the telluranes. The greater weight loss with H_2_O_2_ leaching than with acid etching is due to the higher molecular weight of the telluranes relative to the silicates.

**Figure 5:**
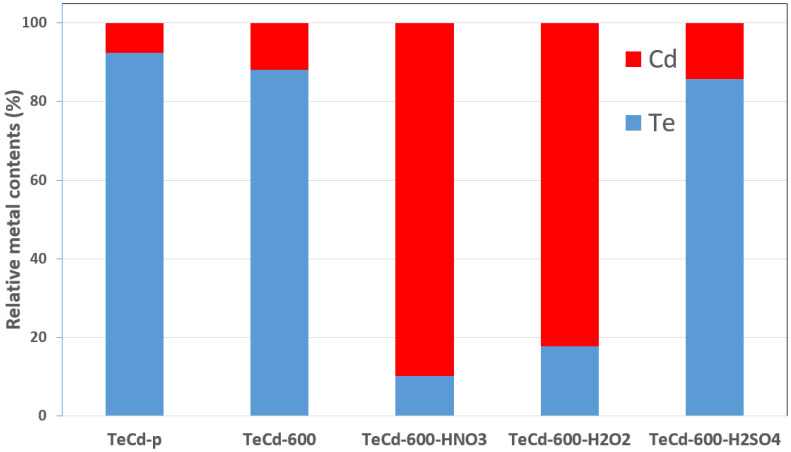
Estimated relative Te and Cd metal atomic contents in the samples based on XPS measurements: before (TeCd-p) and after thermal treatment (TeCd-600), and after leaching for 2h at 50 °C in 1 M H_2_SO_4_ (TeCd-600-H2SO4), 3M HNO_3_ (TeCd-600-HNO3), and 30% H_2_O_2_ (TeCd-600-H_2_O_2_).

Unfortunately, due to the trace amount of cadmium and tellurium in the sample and the surface-sensitive nature of analysis, it is hard to quantify the efficiency of the process at this stage of the research. However, we can undoubtedly note that the leaching process, independently of the used solution, leads to complete CdTe removal from the sample’s surface, and we are able to observe changes in the relative contents of the metals in the sample during the leaching process (Figure 5). On this basis, we can conclude that, in the cadmium removal process, the thermal treatment stage at 600 °C is important, and the most effective Cd removal occurs in sulfuric acid.

Changes observed in the detailed XPS results analysis can help us understand the chemistry of occurring reactions. A more detailed analysis is needed to confirm all the assumptions.

## 4. Conclusions

It was shown herein that highly oxidative solutions such as H_2_O_2_ can be a good choice for the recycling of thin-film modules based on CdTe. XPS measurements confirmed the partial removal of Cd and Te from the surfaces of the tested samples.

According to the presented results, the nature of the chemical reactions that occurred during leaching can be assumed. An improved understanding of the process will aid in the development of new methods for Cd and Te recovery from the obtained solutions.

In future work, a technology for cadmium recovery will be developed that considers the impact on the natural environment and determination of the metal content in the solutions after the etching process. This introductory research was presented at a conference [20]. A research project is planned to use methods other than classical electrolysis to obtain high-purity cadmium from etching solutions.

## Figures and Tables

**Table 1:** Summary of experimental work on metal recovery from recycled thin-film solar modules.

Literature	Type of PV Module	Process	Scale
[4]	CdTe, CIGS (damaged modules, production waste)	Grinding in a hammer mill Abrasion and sieving < 150 µm Flotation Purifying	General
[4]	CdTe, CIGS (whole modules)	Thermal delamination Vacuum dedusting Flotation Purifying	General
[7]	CdTe	HNO_3_ leaching DC electrolysis Solution decomposition	Academic research
[7]	CIGS	HNO_3_ leaching EVA hydrolysis and flotation DC electrolysis Oxidation and distillation of the Cu/Se mixture Solution decomposition	Academic research
[8]	CIGS	Thermal treatment and removal of the glass layer Removal of residual EVA in acetic acid Mechanical cutting of the absorber layer Washing residuals with dilute nitric acid from the glass surface	Academic research
[9]	CdTe	Grinding the module Leaching with a mixture of acid and perhydrol Precipitation of Cd and Te Treatment of enriched sludge after the process Recycling of cleaned glass	Pilot studies
[10]	CdTe	Leaching with HNO_3_ Addition of Ca to precipitate Cd and Te Calcination of the sludge at temperatures below 500 °C Washing with water and drying Calcination to obtain CaO	Pilot studies
[11]	CIS (semiconductor coated glass, production waste)	Grinding Dissolving in oxidizing acid Filtration and separation of the precipitate Liquid–liquid extraction (separation of In from Ga/Se mixture) Purification	Pilot studies
[12]	CdTe	Mechanical disintegration Pyrolysis at temperatures above 400 °C in oxygen Exposure to a mixture of Cl_2_ and N_2_ at 400 °C Cooling and sequential precipitation of CdCl_2_ and TeCl_4_	Academic research
[13]	CdTe	Crushing Washing with H_2_SO_4_+H_2_O_2_ Columns with a chelating agent (Cu) Cation exchange column (Cd and Fe) Electrochemical process (Cd) Precipitated with sodium carbonate (Te)	Academic research

## Data Availability

The data presented in this study are available on request.

## References

[1] Shibasaki M., Warburg N., Springer J., Lombardelli S. Recycling of Thin Film solar modules Life Cycle Assessment case study. Proceedings of the 21st European Photovoltaic Solar Energy Conference.

[2] Marwede M., Berger W., Schlummer M., Mäurer A., Reller A. (2013). Recycling paths for thin-film chalcogenide photovoltaic waste—Current feasible processes. Renew. Energy.

[3] Fthenakis V., Athias C., Blumenthal A., Kulur A., Magliozzo J., Ng D. (2020). Sustainability evaluation of CdTe PV: An update. Renew. Sustain. Energy Rev..

[4] Berger W., Simon F.-G., Weimann K., Alsema E. (2010). A novel approach for the recycling of thin film photovoltaic modules. Resour. Conserv. Recycl..

[5] Klugmann-Radziemska E. (2010). Fotowoltaika W Teorii I Praktyce.

[6] Kuczyńska-Łażewska A., Klugmann-Radziemska E. (2019). Influence of Fragment Size on the Time and Temperature of Ethylene Vinyl Acetate Lamination Decomposition in the Photovoltaic Module Recycling Process. Materials.

[7] Goozner R., Drinkard W., Long M., Byrd C. A process to recycle thin film PV materials. Proceedings of the Conference Record of the Twenty Sixth IEEE Photovoltaic Specialists Conference 1997.

[8] Kushiya K., Tanaka M., Ohshita M. (2003). Development of recycling and reuse technologies for large-area Cu(InGa)Se2-based thin-film modules. 3rd World Conf. Photovolt. Energy Convers..

[9] Sapich G., Weimann K., Berger W., Adam C. Sustainable Recovery of Tellurium and Indium from Thin Film Photovoltaic Modules: EU-LIFE Project Resolved. C:/pdflib/00021774.pdf.

[10] Goozner R.E., Long M.O., Drinkard W.F. (1999). Recycling of CdTe Photovolatic Waste. U.S. Patent.

[11] Schwarze J. (2007). SENSE Report Summary.

[12] Campo M.D., Dieter B., Gegenwart R., Beier J. (2003). Process for Recycling CdTe/CdS Thin Film Solar Cell Modules. U.S. Patent.

[13] Fthenakis V.M., Duby P., Wang W., Graves C., Belova A. Recycling of CdTe Photovoltaic Modules: Recovery of Cadmium and Tellurium. In Poceedings of the 21st European photovoltaic solar energy conference 2006.

[14] Tao J., Yu S. (2015). Review on feasible recycling pathways and technologies of solar photovoltaic modules. Sol. Energy Mater. Sol. Cells.

[15] Aravelli S.G., Ramavathu S.N. (2020). Smart and sustainable technologies for recycling photovoltaic panels. Environ. Chall..

[16] NIST X-ray Photoelectron Spectroscopy (XPS) Database, Version 3.5. https://srdata.nist.gov/xps/.

[17] XPSPEAK 4.1 Download (Free)—XPSPEAK41.exe. https://xpspeak.software.informer.com/4.1/.

[18] Wanger C.D., Riggs W.M., Davis L.E., Moulder J.F., Muilenberg G.E. (1997). Handbook of X-ray Photoelectron Spectroscopy.

[19] Król A., Mazurek T. (1965). Metalurgia Cynku I Kadmu.

[20] Kuczyńska-Łażewska A., Klugmann-Radziemska E. (2021). Influence of the fragment size on the recycling process of thin-film modules based on CdTe. J. Int. Sci. Publ..

